# Effects of gait support in patients with spinocerebellar degeneration by a wearable robot based on synchronization control

**DOI:** 10.1186/s12984-018-0425-4

**Published:** 2018-09-19

**Authors:** Atsushi Tsukahara, Kunihiro Yoshida, Akira Matsushima, Kumiko Ajima, Chika Kuroda, Noriaki Mizukami, Minoru Hashimoto

**Affiliations:** 1 Department of Mechanical Engineering and Robotics, Faculty of Textile Science and Technology, Shinshu University, 3-15-1 Tokida, Ueda, 386-8567 Japan; 2 Institute for Fiber Engineering (IFES), Interdisciplinary Cluster for Cutting Edge Research (ICCER), Shinshu University, Ueda, Japan; 3 Department of Brain Disease Research, Shinshu University School of Medicine, 3-1-1 Asahi, Matsumoto, 390-8621 Japan; 4 Institute for Biomedical Sciences (IBS), Interdisciplinary Cluster for Cutting Edge Research (ICCER), Shinshu University, Matsumoto, Japan; 5 Kakeyu-Misayama Rehabilitation Center Kakeyu Hospital, 1308 Kakeyu, Ueda, 386-0396 Japan; 6 Department of Health Sciences, Graduate School of Medicine, Shinshu University, 3-1-1 Asahi, Matsumoto, 390-8621 Japan

**Keywords:** Spinocerebellar degeneration, Gait support, Synchronization control

## Abstract

**Background:**

Spinocerebellar degeneration (SCD) mainly manifests a cerebellar ataxic gait, leading to marked postural sway and the risk of falling down. Gait support using a wearable robot is expected to be an effective solution to maintaining the status quo and/or delaying symptom progression. The aim of this study was to evaluate the effects of gait support in patients with SCD by using a wearable robotic system called curara ^®;^ while undergoing walking tests.

**Methods:**

The curara system assists both the hip and knee joints and supports the wearer’s rhythmic gait using a synchronization control based on a central pattern generator. The system reflects the wearer’s intended motion in response to the gait support by detecting an interactive force that is generated from slight movements of the wearer. The degree of coordinated motion between the robot and the wearer can be adjusted by modifying the synchronization gain. In this study, we provided gait support using three high-gain conditions (A, B, C) to more easily follow the wearer’s movement in each joint. The synchronization gains for both the hip and knee joints (i.e., *C*_*h*_ and *C*_*k*_) were set at 0.5 for condition A and at 0.4 for condition B. Condition C had different gains for the hip and knee joints (i.e., *C*_*h*_=0.4 and *C*_*k*_=0.5). With the walking test, we assessed the effects of the gait support provided by the curara system on walking smoothness (measured using the harmonic ratio: HR) and spatiotemporal parameters (gait speed, stride length, cadence) in SCD patients (n=12). We compared the performance between the three high-gain conditions and without assistance from the robot.

**Results:**

Under condition C, the HRs in the anteroposterior, mediolateral, and vertical directions (HR-AP, HR-ML, and HR-V) were especially high compared with those under conditions A and B. The results of the statistical analyses using repeated measures analysis of variance followed by Tukey’s test showed that gait support with condition C results in a statistically significant increase in the HR-AP (2.04 ±0.52; *p*=0.025) and HR-V (2.06 ±0.37; *p*=0.032) when compared with walking without assistance from the system. In contrast, the gait speed, stride length, and cadence under condition C were no major changes in most patients, compared with the patient’s walking without assistance.

**Conclusions:**

The significantly increased HR indicates that gait support under condition C achieved smoother walking than when not wearing the power unit of the system. Consequently, we suggest that gait support using the curara system has the potential to improve walking smoothness in patients with SCD.

## Background

Spinocerebellar degeneration (SCD), an intractable neurodegenerative disease, most often develops a slowly progressive cerebellar ataxia. Patients with SCD predominantly suffer from gait disturbance due to the degeneration and loss of nerve cells in the cerebellum, brainstem, spinal cord, and basal ganglia [1, 2]. The cerebellar ataxic gait associated with SCD, therefore, not only shows an increased postural sway but also poses the risk of falling down [3]. Although the rate of disease progression varies with the disease subtype and the individual, the patients are eventually confined to a wheelchair or are bedridden. Such decreased daily activity could then lead to disuse syndrome, including the development of arthrogryposis, muscular atrophy, thromboembolism, and aspiration pneumonia. Curative medical treatment for SCD, unfortunately, has not been reported.

There have been some reports, however, that physical therapy and training within a specified period had a positive effect on the balance and ataxic gait of patients with SCD [4–6]. For instance, intensive gait training for patients with spinocerebellar ataxia types 6 and 31 (SCA6, SCA31) significantly alleviated the ataxia and improved gait speed in the short term and sustained the functional effects over the long term [7]. Hence, maintaining motor functions in the lower limbs via gait training is extremely important for improving their activities of daily living (ADL) and quality of life (QOL).

Gait support using a wearable robot, which helps the wearer walk while actively moving the legs, is an effective solution to facilitate the above-mentioned training and to maintain ADL and QOL in patients with SCD. Until now, various exoskeleton robots have been studied to support walking for patients with stroke and spinal cord injury (SCI) [8–12]. Those studies focused on walking support for patients who find it difficult to stand up and/or walk by themselves. These systems, therefore, have heavy exoskeletal frames to bear the wearer’s weight, and large power units to generate the higher assistive torque. The robot suit HAL ^®;^ supports the gait of patients with neuromuscular disease, stroke, and SCI based on bioelectrical signals (BESs) that are detected from the skin surface just before the corresponding muscle contraction [13–15]. That system might help such patients recover their motor functions, although it is difficult to identify the precise sites on which to place the BES sensors to detect the muscle action potentials of these patients. Recently, walking support systems based on nonlinear oscillator models, instead of detecting the BES, have been developed in the field of wearable robots [16–19]. One of the main advantages of the system is its capacity to adapt to the wearer’s cyclical movement changes. In addition, gait assistance using these models (e.g., adaptive frequency oscillators) allows a wearable robot to assist walking while being synchronized with the wearer’s frequency and phase.

Our research resulted in the development of a wearable robotic system, curara ^®;^, that supports the gait of patients by synchronizing with their motional intention [20–23]. The curara system detects slight movement in the wearer’s lower limbs via a torque sensor (instead of a BES sensor) built into each power unit. The curara’s controller is a synchronization control based on a neural oscillator that is a mathematical model of a central pattern generator (CPG) [24, 25]. The CPG is an oscillatory neuronal network within the spinal cord that produce rhythmic motor patterns without oscillatory inputs from a peripheral sensory feedback and a higher-level of central nerve system [26–29]. This control method generates rhythmic and continuous gait while utilizing the detected movement of the wearer. In addition, the system reflects the intended motion of the wearer by adjusting the synchronization gains, which is a control parameter that allows it to move harmoniously with the wearer. As a preliminary step toward the clinical trial for various SCD patients, we needed to investigate the effects of curara on the gait in the patients with SCD.

The purpose of this study was thus to evaluate the effects of gait support in patients with SCD by applying the curara system and measuring the results with walking tests. This article describes the hardware configurations of the curara system, the gait support method based on the synchronization control using the neural oscillator, and the experimental results of the walking tests for the patients with SCD.

## Methods

### Mechanical design of the wearable robotic system

**Fig. 1:**
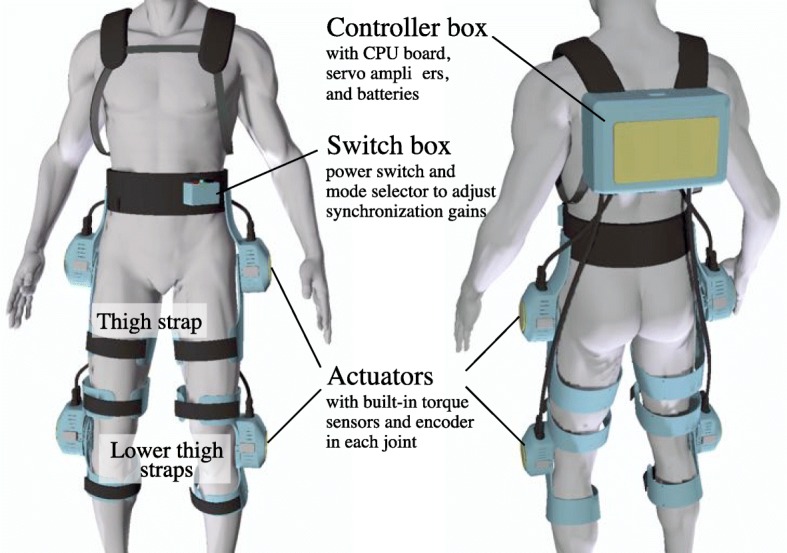
System configuration of a wearable robotic system “curara.” This wearable robotic system was developed to support the wearer’s coordinated motion in the lower limbs. The main components of the curara are actuators, joint supports, a switch box, and a controller box. The total weight of the system is about 5.8 kg. The joint supports can be customized for each subject’s link length because its attachments between the hip joint frame and knee joint frame are composed of Velcro tape, which is a soft material. Actuators are separately attached on the knee and hip joints by the joint supports molded from acrylonitrile butadiene styrene resin without using metallic exoskeleton frames

The wearable robotic system curara, shown in Fig. 1, supports the coordinated motion of the wearer’s hip and knee joints. The main characteristic of the hardware is its wearable structure, which is different from others. Generally, exoskeleton robots rigidly secure the actuators to the wearer’s leg joints using a metallic frame, providing walking support in the sagittal plane. With those robots, however, the wearer is extremely limited regarding voluntary movements in the frontal and horizontal planes because of the frames that connect the actuators in each joint. In contrast, the curara system does not employ a metallic exoskeletal frame [21]. Each actuator is separately attached to the knee and hip joints using joint supporters molded from acrylonitrile butadiene styrene resin. These resin joint supporters are fixed to the mid and lower thigh of the wearer with straps. This structure places less restriction of motion in the frontal and horizontal planes than the exoskeleton structure, and it does not require an adjustment mechanism of the link length for fitting the misalignment of the rotation center between the wearer’s joint and the curara’s actuator. The actuator is composed of an axial gap-type motor (Sanyo Denki Co., Ltd., Japan) with a HarmonicDrive ^®;^ reducer (Harmonic Drive Systems Inc., Japan), a built-in encoder, and torque sensors. It has a rated power of about 30 W, a rated torque of about 7 Nm, and a mass of about 480 g (without the cover). The torque sensor consists of three strain gauges fixed on the flex-spline of the harmonic drive gear and a flexible printed circuit. The elasticity of the flex-spline is used to detect the interactive force, which is a strain change generated between the wearer’s movement and curara’s movement, because it has a flexible structure made from steel. The fundamental principle of the torque sensing technique has reported in [30]. The controller box, which contains a central processing unit board, batteries, motor drivers, and electrical circuits, is located on the wearer’s back. The switch box has two functional switches: a power supply switch and a switch for adjusting the synchronization gain. The adjustment of the synchronization gain allows the curara system to provide the appropriate gait support that matches to the wearer’s intended motion.

### Synchronization control for gait support

It has been widely accepted that rhythmic locomotion in vertebrate animals, including mammals, is based on the activity of the CPG, which exists in the neuronal circuits within the spinal cord [26]. The CPG produces periodic and alternating motor patterns (e.g., flexion and extension) via excitation and inhibition of interneurons in the central nervous system. Several studies have reported that the spinal circuits in patients with SCI may produce a rhythmic muscle activation pattern for locomotion closely similar to CPG-induced responses [27–29]. These findings suggest that CPG in humans likely exists throughout the lumbar and upper sacral spinal cord. In the robotics field, the CPG model has also been utilized to generate flexible, rhythmic walking using the bipedal robot [31–33]. The control scheme of the curara system, which we designed, is based on the concept of CPG, thereby supporting rhythmic walking of patients with SCD.

**Fig. 2:**
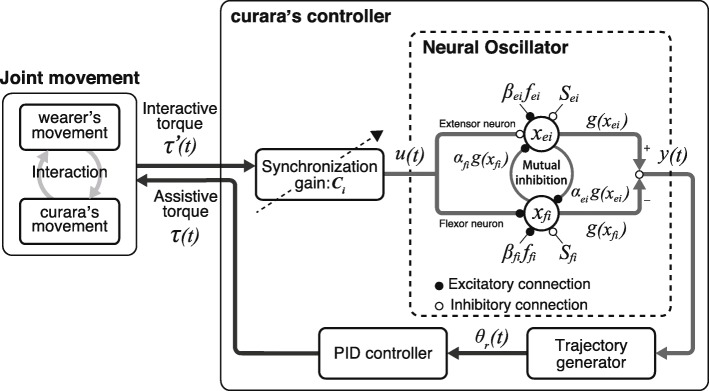
Control scheme of the curara system for gait support. The curara system supports the wearer’s gait using a synchronization control method that uses a neural oscillator based on a central pattern generator. Adjusting the synchronization gain *C*_*i*_ helps curara to assist harmoniously with the wearer’s motion

Figure 2 shows the control scheme of the curara system for gait support. The control algorithm is based on synchronization control utilizing a neural oscillator. The neural oscillator mathematically models the behavior of the CPG as follows:

1 $$\begin{array}{@{}rcl@{}} T_{r} \frac{dx_{\{e, f\}i}}{dt}+x_{\{e, f\}i} &=& - \sum\limits_{ei \neq fi}^{n} \alpha_{\{e, f\}i} \mathrm{g}(x_{\{e, f\}i}) + S_{\{e, f\}i}  \\ &&- \beta_{\{e, f\}i}f_{\{e, f\}i} + u(t)_{,} \end{array} $$

2 $$\begin{array}{@{}rcl@{}}  u(t) &=& \left\{ \begin{array}{ll} \hspace{3mm} C \tau^{'}(t) \hspace{5mm}& ({if} \hspace{2mm} x_{ei}) \\ - \hspace{0.5mm}C \tau^{'}(t) & ({if} \hspace{2mm} x_{fi}) \\ \end{array} \right. \end{array} $$

3 $$\begin{array}{@{}rcl@{}}  T_{a}\frac{df_{\{e, f\}i}}{dt}+f_{\{e, f\}i} &=& \mathrm{g}(x_{\{e, f\}i})_{,} \end{array} $$

4 $$\begin{array}{@{}rcl@{}}  \mathrm{g}(x_{\{e, f\}i}) &=& \text{max}(0,x_{\{e, f\}i})_{,} \end{array} $$

5 $$\begin{array}{@{}rcl@{}}  y(t) &=& g(x_{ei}) - g(x_{fi})_{,} \end{array} $$

$$\begin{array}{@{}rcl@{}} (i &=& 1,2, \ldots,n)_{,}  \end{array} $$

where *x* is the state variable that denotes the membrane potential of the neuron; *α* is the strength of the neuronal inhibitory connection; g is the output variable from each of the neurons that constitute the neural oscillator; *S* is the stationary input to the neuron; *β* is the strength of self-inhibition of the neuron; *f* is the state variable that denotes the degree of self-inhibition of the neuron; *T*_*r*_ and *T*_*a*_ are the time constants of the membrane potential and self-inhibition, respectively; and the subscripts *ei* and *fi* denote the extensor and flexor neurons of *i*-th, respectively. Once the parameters and the initial states of neurons are defined, a cyclical sine-waveform signal *y*(*t*) is generated when the two neurons are alternately activated. The parameter values used in this study are given as follows: *α*_{*e*,*f*}*i*_ = 1.2, *S*_{*e*,*f*}*i*_ = 2.0, and *β*_{*e*,*f*}*i*_ = 2.5. The initial values of *T*_*r*_ and *T*_*a*_ were 0.12 and 0.6, respectively, although these time constants are modified according to the severity of symptoms associated with gait disturbance.

The neural oscillator allows mutual entrainment, synchronizing self-excitation vibration of the output to an external input signal [25]. In this study, the interactive torque $\phantom {\dot {i}\!}\tau ^{\prime }(t)$ multiplied by a synchronization gain *C* is provided to the neural oscillator as the input signal *u*(*t*). The interactive torque is generated from the slight deviation between the wearer’s movement and curara’s movement. It is detected using the torque sensors embedded in the actuator in each joint.

**Fig. 3:**
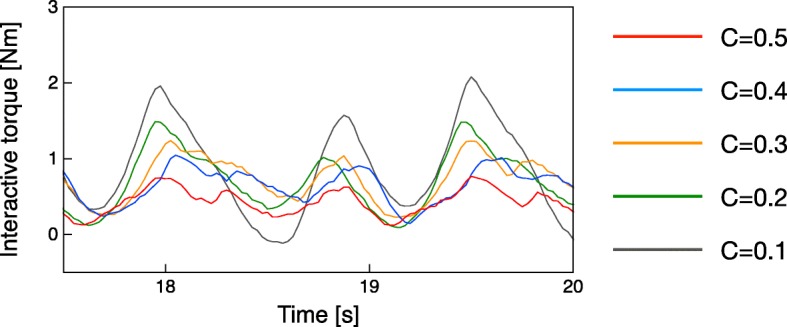
Variations of interactive torque vis-a-vis synchronization gain during gait support in a healthy control subject. The interactive torque, which is detected by motional deviation between curara and the wearer, is increased according to the decrease in the synchronization gain

The synchronization gain setting adjusts the degree of the mutual entrainment in the curara system. Our previous research showed that the output behaviors from the neural oscillator change according to the synchronization gain setting [34, 35]. Low-synchronization gain (e.g., *C* = 0.1) produces stable self-excitation vibration as the output signal from the neural oscillator. Mutual entrainment prominently appears when the synchronization gain is at a higher setting (e.g., *C* = 0.5). Based on these findings, the curara system allows the gain to be adjusted within a range of 0.1-0.5, at intervals of 0.1, using the switch box shown in Fig. 1. Figure 3 shows evidence of interactive torque when the curara system supported the walking of a healthy control subject for each synchronization gain setting. These results show that the amount of interactive torque, which is generated by the motional deviation between the wearer and the curara, decreases with the increasing gain and, conversely, increases with the decreasing gain. These phenomena indicate that a higher synchronization gain allows the rhythmic gait that assists the curara to follow easily the wearer’s movement in each joint. In contrast, a lower gain helps to actively assist the gait with robot-driven movement, although the system is less likely to synchronize with the wearer’s movement.

The output signal $\vec {y}(t)$ from the neural oscillator, which is synchronized with the wearer’s movement, is converted to the desired trajectory $\vec {\theta }_{r}(t)$ for each joint via the trajectory generator as follows:

6 $$\begin{array}{@{}rcl@{}} {} \vec{\theta}_{r}(t) &=& A \vec{y}(t) + B, \end{array} $$

7 $$\begin{array}{@{}rcl@{}} A &\,=\,& \frac{\vec{\theta}_{max}(t) \,-\, \vec{\theta}_{min}(t)}{2}_{,} \hspace{2mm} \text{and} \hspace{2mm} B \,=\, \frac{\vec{\theta}_{max}(t) \,+\, \vec{\theta}_{min}(t)}{2}_{,} \end{array} $$

where *A* is a conversion factor to convert output signal from the neural oscillator into angular amplitude. $\vec {\theta }_{max}(t)$ and $\vec {\theta }_{min}(t)$ are the maximum flexion and maximum extension angles, respectively. *B* is an offset value of each joint to generate asymmetric waveform between flexion and extension directions. The system calculates the desired trajectory based on the output signal from the neural oscillator while assisting gait of wearer. Therefore, it is unnecessary to prepare the unique trajectories required for gait support.

The curara system controls each actuator with a proportional-integral-derivative (PID) control. The feedback controller, like the PID control method, calculates an assistive torque *τ*(*t*) so that a joint angle can follow the desired trajectory, which changes over time. The control law is calculated as follows:

8 $$\begin{array}{@{}rcl@{}}  \vec{\tau}(t) = \vec{K}_{P}\vec{e}(t) + \vec{K}_{I} {\int}^{t} \vec{e}(t)dt + \vec{K}_{D}\vec{\dot{e}}(t)_{,} \end{array} $$

where $\vec {\tau }(t)$ is the command torque in each joint; and $\vec {K}_{P} $, $\vec {K}_{I} $, and $\vec {K}_{D}$ are the PID gains. The single-column matrices $\vec {e}(t)=\vec {\theta }_{r}(t)-\vec {\theta }(t)$ and $\vec {\dot {e}}(t)=\vec {\dot {\theta _{r}}}(t)-\vec {\dot {\theta }}(t)$ represent the displacement errors for the angle and the angular velocity, respectively.

### Participants

Walking tests for patients with SCD in this study were conducted at the Shinshu University School of Medicine, Japan. Inclusion criteria were (1) a definitive diagnosis of SCD including olivopontocerebellar atrophy (OPCA), (2) relatively pure cerebellar ataxia, and (3) ability to walk independently with a slight stagger. A total of 12 individuals (6 men, 6 women) fulfilled the criteria and were recruited for the walking tests. Sex, age, age at onset, duration of disease, and SCD subtype are shown in Table 1. The diagnosis of SCA6 and SCA31 was confirmed by genetic testing. The severity of cerebellar ataxia was assessed in each patient using the Scale for the Assessment and Rating of Ataxia (SARA) [36, 37]. The range of SARA total score and SARA gait score in the 12 participants were from 6 to 15 (mean ±standard deviation; 9.8 ±2.3) and from 2 to 3 (mean ±standard deviation; 2.8 ±0.4), respectively.

**Table 1:** Characteristics of patients with spinocerebellar degeneration

Case ID	Sex	Age	Age onset	Duration disease	Subtype	SARA score (gait/total)
1	M	50	34	16	SCA6	3/8
2	M	65	56	9	SCA31	2/8
3	F	72	59	13	SCA31	3/13
4	M	64	53	11	SCA31	3/15
5	F	50	37	13	SCA6	3/10.5
6	F	48	35	13	SCA6	3/10
7	M	66	58	8	SCA31	3/6
8	F	65	43	22	SCA6	3/10
9	F	59	33	26	SCA6	3/9.5
10	F	56	39	17	CCA	3/7.5
11	M	53	51	2	OPCA	3/10
12	M	65	61	4	OPCA	3/9.5

### Experimental procedures

The objective of the experiment is to evaluate the effects of the gait support using the curara system in the patients with SCD during the walking tests.

Patients with SCD first walked along 8 meters of flat ground before being outfitted with the curara system. Next, the parameters, *T*_*r*_ and *T*_*a*_, for the neural oscillator model shown in Eqs. (1) and (3), which determine the amplitude and cycle of the reference trajectories, were extracted from the basic gait data using a three-dimensional motion capture system with 12 Kestrel cameras (MAC3D System, Motion Analysis Co., USA). The patients then walked along the same path but with the support of the curara system. The relations between the interactive torque and the synchronization gain are shown in Fig. 3. It can be seen that this study used a high gain setting so the system could support the SCD patient’s gait with the wearer-driven movement. Table 2 shows the three synchronization condition settings that are utilized in this study. Specifically, the condition C had a different synchronization gain between hip joints and knee joints.

**Table 2:** Condition settings of synchronization gains

Condition settings	Synchronization gains	
	Hip joints	Knee joints
A	0.5	0.5
B	0.4	0.4
C	0.4	0.5

**Fig. 4:**
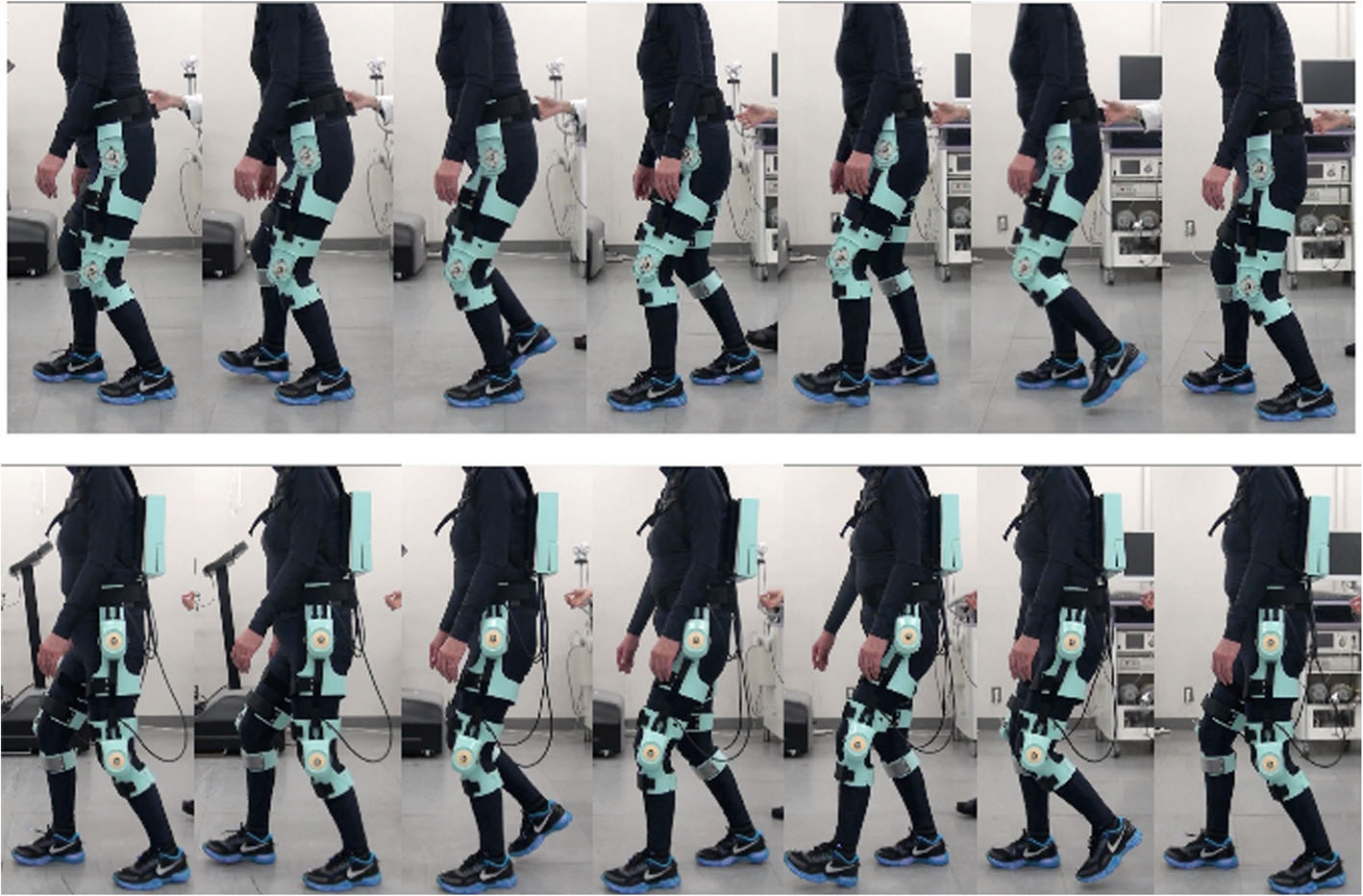
Sequential photographs of a SCD patient without and with gait support during walking tests. Top panels show a patient’s gait before being attached to the power units at each joint. When patients walk without gait support from the curara system, they continue to wear the joint supports molded from deformable plastic. These structures do not disturb the patient’s natural gait because they are completely disconnected from the power unit, so there is no assistive torque. This condition is thus equivalent to not wearing the assistive robot. Bottom panels show the patient’s gait while walked after being outfitted with the curara system, which provides gait support

**Fig. 5:**
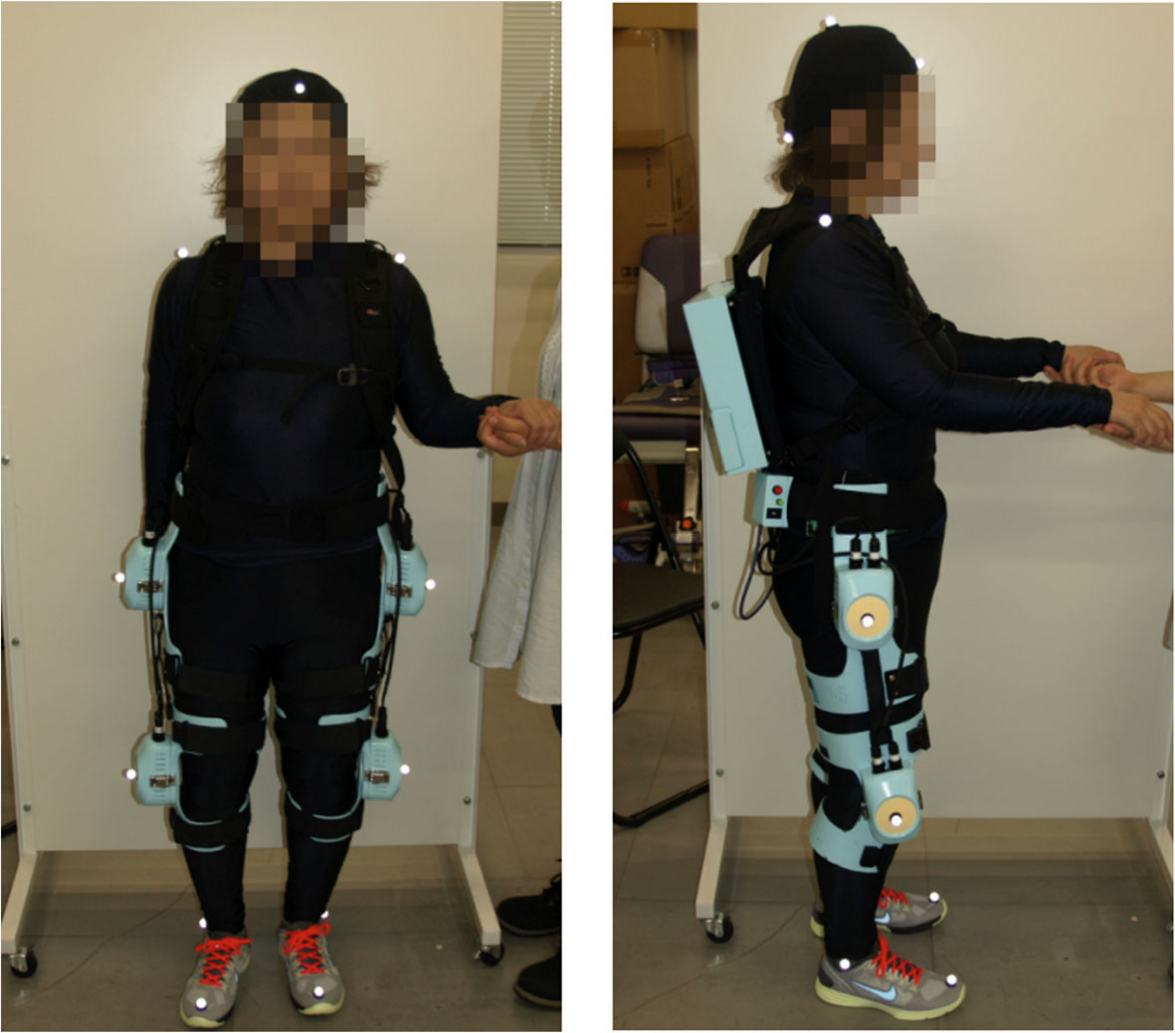
Experimental settings for measuring the gait of patients with spinocerebellar degeneration (SCD). A total 13 points of reflective markers are placed on the wearer’s body to capture the gait using a three-dimensional motion capture system

All walking tests—viz., walking without wearing the power unit (PU) including actuators, a switch box, and a controller box of the curara system (see Fig. 4 on the top), and the gait support with the system under the three conditions (see Fig. 4 on the bottom) —were conducted five times for each trial. Since changes in inertia might affect the gait results of the patients, we disconnected the PU from the lower limb joints of patients to minimize the effects of inertia when the patients walked without using gait support from the curara system. Reflective markers were attached on the patient and curara, as shown in Fig. 5, to record the motion of the gait using motion capture. In addition, a triaxial accelerometer (Wireless Motion Recorder mini, MVP-RF8-GC, MicroStone Co., Ltd., Japan) was attached on the participant’s back to measure the acceleration data for the trunk during walking. To allow ample recovery of the patients, breaks were given frequently, depending on the physical condition of the patient during each test. The walking tests were conducted under the supervision of a medical doctor and the researchers to prevent an unexpected fall.

The walking tests were performed in accordance with all procedures and approved by the Ethics Committee of Shinshu University School of Medicine (No. 2699) under the title of “Clinical application of robotic wear (Curara) for patients with neuromuscular disorders (UMIN000013721, April 15, 2014).” The patients provided informed consent before participating in this test.

### Data analysis

### *Evaluation indexes*

Recorded data using motion capture was postprocessed using the operation software Cortex (MAC3D System; Motion Analysis Corp., Santa Rosa, CA, USA). It was then exported to the analytical software KineAnalyzer (Kissei Comtec Co., Ltd., Matsumoto, Japan), which not only estimates heel-floor contact based on the marker’s position on the ankle but also extracts typical spatiotemporal gait parameters for gait assessment. In this study, four assessment metrics were used: gait speed, cadence, stride length, and the harmonic ratio (HR). The HR, which has been utilized extensively to quantify walking smoothness, walking rhythmicity, and dynamic stability [38–40]. Higher values of the HR indicate a smoother, more stable walking pattern [41]. The HRs in the anteroposterior (AP), mediolateral (ML), and vertical (V) directions (i.e., HR-AP, HR-ML, and HR-V) are calculated from a Fourier analysis of trunk acceleration data at each gait cycle. This study divided the accelerations based on the heel-floor contact, detected by KineAnalyzer software, and then calculated the HRs in each direction. These evaluation indexes were compared with the data collected when walking without wearing the PU of the curara system.

### *Statistical analysis*

All data are expressed as means and ± standard deviations (SD). Statistical analyses were conducted in all gait parameters (i.e., mean HRs, gait speed, cadence, stride length). In this study, repeated measures analysis of variance (ANOVA) and Tukey’s test were used to compare gait results when not wearing the PU of the curara system versus when receiving gait assistance under the three conditions shown in Table 2. A *p*-value <0.05 was considered to indicate statistical significance. These analyses were performed using R software (Foundation for Statistical Computing, Vienna, Austria).

## Results

### Harmonic ratio

Table 3 shows the mean HR ± SD of the patients in the AP, ML, and V directions during the walking tests. The results of HR-AP, HR-ML, and HR-V not wearing the PU of the curara system were 1.50 ±0.30, 1.49 ±0.19, and 1.63 ±0.29, respectively. The results using curara system’s gait support with the synchronization gain conditions were as follows: for condition A: HR-AP=1.68 ±0.41, HR-ML=1.52 ±0.31, and HR-V=1.70 ±0.43, respectively; for condition B: HR-AP=1.79 ±0.52, HR-ML=1.53 ±0.35, and HR-V=1.84 ±0.35, respectively; and for condition C: HR-AP=2.04 ±0.52, HR-ML=1.67 ±0.50, and HR-V=2.06 ±0.37, respectively. The HRs in all directions when the synchronization gain condition C was used, were higher than those for the gait support under either the A or B condition. In particular, the HR-AP and HR-V under gait support using the synchronization gain condition C were statistically significantly higher than those obtained while not wearing the PU (HR-AP: *p*=0.025, HR-V: *p*=0.032, where *p*<0.05 indicates a statistically significant difference).

**Table 3:** Mean harmonic ratio (HR) ± standard deviation (SD)

HR	Without PU	Cond. A		Cond. B		Cond. C	
		Mean ± SD	*p*-value	Mean ± SD	*p*-value	Mean ± SD	*p*-value
*H* *R*_*AP*_	1.50 ±0.30	1.68 ±0.41	0.745	1.79 ±0.52	0.389	**2.04 ±0.52** ^∗^	0.025
*H* *R*_*ML*_	1.49 ±0.19	1.52 ±0.31	0.998	1.53 ±0.35	0.993	1.67 ±0.50	0.636
*H* *R*_*V*_	1.63 ±0.29	1.70 ±0.43	0.964	1.84 ±0.35	0.487	**2.06 ±0.37** ^∗^	0.032

Synchronization gain conditions A, B, and C are shown in Table 2. In the tables, Bold numbers indicate a significant difference (* *p*<0.05, determined by repeated measures analysis of variance (ANOVA) followed by Tukey’s test) between the patients’ gait without wearing the power unit (PU) including actuators, a switch box, and a controller box of the curara system

### Spatio-temporal parameters

Tables 4, 5, and 6 show the results of spatiotemporal parameters throughout the walking tests for 12 patients with SCD. Under the synchronization gain setting conditions, we found that the gait speed, stride length, and cadence in a few patients with SCD were significantly increased compared with the results when not wearing the PU of the system. However, the gait support with the system under the gain conditions did not provide conclusive proof that these spatiotemporal parameters could be improved in all SCD patients.

**Table 4:** Observed mean gait speed ± standard deviation (SD)

Case ID	Gait speed ± SD [m/s]			
	Without PU	Cond. A	Cond. B	Cond. C
1	1.05 ±0.02	1.07 ±0.03	1.08 ±0.01	1.11 ±0.02
2	0.64 ±0.08	0.47 ±0.08	0.52 ±0.02	0.62 ±0.07
3	0.70 ±0.09	0.94 ±0.04	0.80 ±0.07	**1.00 ±0.02** ^∗^
4	0.65 ±0.12	0.66 ±0.03	0.65 ±0.06	0.68 ±0.03
5	0.73 ±0.11	0.65 ±0.12	0.75 ±0.03	0.73 ±0.03
6	1.12 ±0.07	0.87 ±0.09	0.86 ±0.02	0.89 ±0.03
7	1.15 ±0.01	1.06 ±0.07	1.13 ±0.06	**1.26 ±0.04** ^∗^
8	0.48 ±0.06	0.35 ±0.10	0.56 ±0.03	0.58 ±0.02
9	0.37 ±0.04	**0.49 ±0.08** ^∗^	**0.60 ±0.02** ^∗∗^	**0.57 ±0.03** ^∗∗^
10	0.83 ±0.06	0.78 ±0.07	0.87 ±0.05	**0.99 ±0.08** ^∗^
11	0.92 ±0.02	0.81 ±0.08	0.92 ±0.03	0.98 ±0.01
12	0.92 ±0.10	0.93 ±0.11	0.98 ±0.05	0.92 ±0.04

Bold numbers show the statistically increase of gait speed compared with the patient’s basic gait without wearing the power unit (PU) including actuators, a switch box, and a controller box of the curara system. ^∗^*p*<0.05, ^∗∗^*p*<0.001

**Table 5:** Observed mean stride length ± standard deviation (SD)

Case ID	Stride length ± SD [cm]			
	Without PU	Cond. A	Cond. B	Cond. C
1	128.08 ±0.13	123.56 ±1.79	127.99 ±1.04	126.54 ±1.49
2	60.83 ±10.38	46.42 ±6.85	48.82 ±1.74	58.21 ±6.86
3	81.83 ±6.10	100.20 ±4.79	89.75 ±1.09	**104.39 ±6.55** ^∗^
4	64.16 ±7.51	62.13 ±1.84	62.85 ±3.95	63.69 ±1.79
5	83.51 ±10.87	76.90 ±10.86	89.85 ±5.98	87.27 ±3.90
6	107.76 ±6.03	91.27 ±4.58	95.48 ±1.91	95.90 ±1.38
7	128.34 ±2.54	129.09 ±5.55	130.38 ±5.66	**138.45 ±4.18** ^∗^
8	58.84 ±6.09	46.90 ±7.42	63.46 ±3.36	64.07 ±1.84
9	59.09 ±7.36	61.97 ±10.44	74.99 ±3.61	67.66 ±3.81
10	93.79 ±12.63	91.41 ±4.37	100.16 ±4.98	**107.32 ±0.78** ^∗^
11	117.84 ±2.30	104.84 ±4.84	110.24 ±3.64	115.63 ±1.30
12	104.75 ±8.91	104.09 ±10.00	111.13 ±6.87	105.84 ±3.98

Bold numbers show the statistically increase of stride length compared with the patient’s basic gait without wearing the power unit (PU) including actuators, a switch box, and a controller box of the curara system. ^∗^*p*<0.05, ^∗∗^*p*<0.001

**Table 6:** Observed mean cadence ± standard deviation (SD)

Case ID	Cadence ± SD [steps/min]			
	Without PU	Cond. A	Cond. B	Cond. C
1	98.41 ±2.30	103.69 ±0.97	101.72 ±1.84	105.20 ±3.10
2	127.70 ±6.74	122.26 ±4.89	127.20 ±1.77	126.96 ±1.16
3	102.80 ±6.25	112.35 ±9.69	106.51 ±10.66	115.53 ±4.66
4	120.39 ±8.87	128.25 ±3.07	123.56 ±5.18	127.94 ±3.20
5	104.35 ±5.99	100.51 ±4.36	100.02 ±3.26	99.78 ±0.67
6	124.53 ±3.81	114.50 ±7.31	108.59 ±4.97	111.48 ±4.36
7	107.77 ±2.23	98.70 ±2.37	103.72 ±5.69	109.36 ±2.35
8	97.91 ±2.64	89.35 ±1.97	106.37 ±0.18	108.80 ±1.56
9	74.53 ±4.73	**95.65 ±2.76** ^∗∗^	**96.76 ±4.17** ^∗∗^	**100.89 ±3.40** ^∗∗^
10	106.41 ±7.44	102.21 ±5.46	104.03 ±4.24	110.44 ±8.14
11	93.57 ±0.32	92.55 ±5.30	**99.85 ±1.16** ^∗^	**102.02 ±1.20** ^∗∗^
12	105.24 ±4.32	107.09 ±5.26	106.20 ±2.70	104.65 ±2.59

Bold numbers show the statistically increase of cadence compared with the patient’s basic gait without wearing the power unit (PU) including actuators, a switch box, and a controller box of the curara system. ^∗^*p*<0.05, ^∗∗^*p*<0.001

## Discussion

We evaluated the effects of gait support in patients with SCD that resulted from applying the curara system during walking tests. Several studies previously investigated the characteristic ataxic gait of patients with SCD in clinical practice [42–44]. These previous findings quantitatively assessed the usefulness of a triaxial accelerometer during a 10-m walking test in SCD patients compared with subjects without gait impairment. In contrast, the present study provides the first results indicating that the gait support provided by the wearable robotic curara system retains the potential to effect of the patients’ gait function such as walking smoothness and spatiotemporal parameters.

The walking test results for the 12 patients with SCD showed that the mean HRs during the gait support using the synchronization gain condition C were significantly improved in the AP and V directions compared with the results when the patients walked without wearing the PU (which included the actuators, controller box, and switch box of the curara system). These results are similar to the variability characteristic of HR related to the risk for fall while walking. A rise in the HR value means that the subject’s walking results in a smoother, more stable gait pattern [38–41]. In previous studies, the HR obtained from acceleration during walking was clinically useful for estimating the risk of falling [45, 46]. Menz *et* *al*., showed that subjects at high risk of falling walked with decreasing HRs in the AP and V directions compared with young control subjects and older subjects without balance problems [45]. Doi *et* *al*., reported that, not only the HRs in the AP and V directions were lower in those more likely to fall (i.e., experiencing a fall at least once a year) than in non-fallers, but the HR in the V direction indicated a risk of falling [46]. Based on these findings, the significantly increased HRs in the AP and V directions in the present study indicated that gait support with gain condition C might lead to a lower risk of falling than with the patient’s basic gait not wearing the PU. In contrast, the gait speed, stride length, and cadence under the gain condition C showed significant increases only in 4, 3, and 2 of the 12 patients, respectively. Thus, the spatiotemporal parameters with gait support using the curara system did not change in most patients with SCD when compared with the results achieved when not wearing the PU.

We conducted preliminary experiments on some patients with SCD to determine the optimal synchronization gains before administering the walking test in this study. As a result, we selected conditions A and B for the study. The gait support under these conditions in the preliminary experiments increased the HRs of participants compared with other conditions. We also added condition C to the study based on the standpoint of motion dynamics. In general, a swing motion of the lower thigh was added based on an inertial force due to a compound pendulum motion around the hip joint axis [47]. That is, the moment of force during biped walking, which is generated around the hip joint axis, is greater than that around the knee joint axis. In the setting of gain condition C, as shown in Table 2, the synchronization level of the hip joint is lower than that of the knee joint, unlike with the settings of conditions A and B. This combination of the synchronization gain indicates that more assistive torque is produced from the curara’s actuator at the hip joint than at the knee joint. As a result, the gait support from the curara system with condition C, which has dynamic characteristics similar to those of the natural walking of humans, showed the highest effects of gait in patients with SCD.

In the control strategy of this study, the curara system supported a rhythmic gait for patients with SCD by the synchronization control method that uses a neural oscillator based on a CPG. The synchronization gain settings were applied at higher values to support the patient’s gait with the wearer-driven movement based on the outcomes in our previous study [34, 35]. In addition, the parameters of the neural oscillator model, which determine the amplitude and cycle of the reference trajectories in each joint, were extracted from the basic gait data obtained when the patient was not wearing the PU of the system. These data were utilized in the control algorithm in unchanged states. In contrast, the gait support of the curara system in stroke patients—which provided larger amplitude and faster cycles (as the reference trajectories) than those with the basic gait—accounted for the significant increases in their gait speed, stride length, and cadence [23]. Given these results, it is expected that increased gait speed, stride length, and cadence in patients with SCD might be obtained by realigning the parameters.

Gait support with the synchronization-based control of the curara system in the present study positively affected the HR in patients with SCD. From the standpoint of the risk of falling while walking, our study could potentially play an important role in the fields of rehabilitation and patient welfare as the conventional wearable robots have been used to just assess the patient’s gait based on the general parameters (e.g., gait speed, cadence, and stride length). On the other hand, it has been reported that the spatiotemporal parameters and balance during walking are dependent on gait speed [48]. The relation with gait speed vis-a-vis the improvement of the HR during gait support is still unclear as the limitations of this study include the fact that the number of participants is small and the walking tests were performed for only a short period. Thus, future studies will require realistic tests with more subjects for regular and longer times to clarify these issues and to evaluate the feasibility of clinical application. We are currently developing a balance control algorithm for SCD patients that we expect will help patients reconstruct their gait balance function using the curara system.

## Conclusions

In this study, we evaluated the effects of gait support in 12 patients with SCD in regard to not wearing the PU of the curara system and wearing it during a walking exercise. Significant improvements in the HR indicated that the gait support under gain condition C achieved smoother walking than when not wearing the PU. Although gait speed, stride length, and cadence in a few patients significantly increased compared with the results when not wearing the PU, the effects of the gait support system under the three gain conditions were inconclusive regarding whether it improved these spatiotemporal parameters in all patients. Consequently, we suggest that the gait support with synchronization-based control using the curara system has the potential to improve walking smoothness in patients with SCD.

## Abbreviations

AP: Anteroposterior
ANOVA: Analysis of variance
ADL: Activities of daily living
BES: Bioelectrical signal
CPG: Central pattern generator
HR: Harmonic ratio
ML: Mediolateral
OPCA: Olivopontocerebellar atrophy
PID: Proportional-integral-derivative
PU: Power unit
QOL: Quality of life
SCI: Spinal cord injury
SCD: Spinocerebellar degeneration
SCA6: Spinocerebellar ataxia type 6
SCA31: Spinocerebellar ataxia type 31
SARA: Scale for the assessment and rating of ataxia
SD: Standard deviation
V: Vertical
